# *Spiroplasma* Isolated From Third-Generation Laboratory Colony *Ixodes persulcatus* Ticks

**DOI:** 10.3389/fvets.2021.659786

**Published:** 2021-03-26

**Authors:** Alexandra Beliavskaia, Vaclav Hönig, Jan Erhart, Tereza Vyhlidalova, Martin Palus, Jiri Cerny, Irina Kozlova, Daniel Ruzek, Ana M. Palomar, Lesley Bell-Sakyi

**Affiliations:** ^1^Department of Infection Biology and Microbiomes, Institute of Infection, Veterinary and Ecological Sciences, University of Liverpool, Liverpool, United Kingdom; ^2^Institute of Parasitology, Biology Centre of the Czech Academy of Sciences, Ceske Budejovice, Czechia; ^3^Veterinary Research Institute, Brno, Czechia; ^4^Centre for Infectious Animal Diseases, Faculty of Tropical AgriSciences, Czech University of Life Sciences, Prague, Czechia; ^5^Science Center for Family Health and Human Reproduction Problems, Irkutsk, Russia; ^6^Centre of Rickettsiosis and Arthropod-Borne Diseases, Hospital Universitario San Pedro-CIBIR, Logroño, Spain

**Keywords:** tick cell line, endosymbiont, *Spiroplasma*, tick colony, *Ixodes persulcatus*

## Abstract

*Spiroplasma* are vertically-transmitted endosymbionts of ticks and other arthropods. Field-collected *Ixodes persulcatus* have been reported to harbour *Spiroplasma*, but nothing is known about their persistence during laboratory colonisation of this tick species. We successfully isolated *Spiroplasma* from internal organs of 6/10 unfed adult ticks, belonging to the third generation of an *I. persulcatus* laboratory colony, into tick cell culture. We screened a further 51 adult male and female ticks from the same colony for presence of *Spiroplasma* by genus-specific PCR amplification of fragments of the 16S rRNA and *rpoB* genes; 100% of these ticks were infected and the 16S rRNA sequence showed 99.8% similarity to that of a previously-published *Spiroplasma* isolated from field-collected *I. persulcatus*. Our study shows that *Spiroplasma* endosymbionts persist at high prevalence in colonised *I. persulcatus* through at least three generations, and confirms the usefulness of tick cell lines for isolation and cultivation of this bacterium.

## Introduction

Ixodid ticks naturally harbour a variety of bacterial symbionts that may be obligately or facultatively intracellular and are transovarially transmitted. These include species of the genera *Rickettsia, Coxiella, Midichloria* and *Spiroplasma* that occur with high frequency (1–4) and less common or well-characterised species of the genus *Francisella* (1, 3) and *Occidentia* (5). The insect symbionts *Cardinium, Wolbachia, Arsenophonus* and *Rickettsiella* have also been detected in or isolated from ticks (3, 6–10) but it is unclear whether or not their presence results from parasitism by insects such as the wasp *Ixodiphagus hookeri* (7, 9) or cohabiting mites (author's unpublished observations), and they are not known to be transovarially transmitted in ticks. Most studies of occurrence of bacterial symbionts in ticks are based on molecular detection in DNA extracted from individual or pooled ticks sampled directly from the field. Some recognised or putative tick symbionts have been isolated into culture, in either mammalian or tick cells; these include several species of *Rickettsia* (11–16), *Francisella* (17), several strains of *Spiroplasma* (10, 18–21) and one isolate each of *Arsenophonus, Occidentia* and *Rickettsiella* (5, 8, 10). In all cases, the unfed or partially-fed ticks had been collected from the field, and bacteria were isolated directly from homogenised/macerated whole ticks or aseptically-dissected internal organs, or from eggs laid by engorged female ticks.

*Ixodes persulcatus*, a tick species distributed widely from the eastern Baltic coast to Japan (22–25), has been reported to harbour symbionts including the Montezuma agent, now called *Candidatus* Lariskella arthropodarum (26–28), *Coxiella* and *Spiroplasma* spp. (29), as well as human and livestock pathogens including tick-borne encephalitis virus (TBEV), Kemerovo virus, Alongshan virus, *Anaplasma phagocytophilum, Candidatus* Neoehrlichia mikurensis, *Ehrlichia muris, Rickettsia helvetica, Rickettsia heilongjiangensis, Candidatus* Rickettsia tarasevichiae, *Borrelia miyamotoi, Borrelia burgdorferi* sensu lato, *Theileria equi* and several species of *Babesia* (25, 30–38). Co-infections with multiple pathogens and symbionts are common (29, 35, 39). There is a single report of isolation into culture of bacterial symbionts from Japanese *I. persulcatus*: *R. helvetica* and a *Spiroplasma* were isolated from field-caught adult male ticks into an *Ixodes scapularis* cell line (10).

Research on transmission of tick-borne pathogens of medical and veterinary interest depends largely on ticks maintained in laboratory colonies. However, few studies have assessed such ticks for presence of symbionts, despite the potential influence of the latter on the ability of ticks to harbour (40) and/or transmit pathogens. Prevalence of *Candidatus* Midichloria mitochondrii determined by molecular methods was found to be lower in *Ixodes ricinus* ticks from laboratory colonies than in field ticks, and to decrease (albeit in a small sample size) with increasing numbers of tick generations (2). A subsequent study, using a more sensitive assay, revealed the presence of extremely low levels of *Ca*. M. mitochondrii DNA in 60% of >10th generation laboratory colony *I. ricinus* (41). *Ixodes arboricola* were screened for bacterial symbionts by PCR and higher incidences were found in field-collected ticks than in laboratory colony ticks of three genera: *Rickettsiella* (28.0 vs. 0%), *Midichloria* (1.3 vs. 0%) and *Spiroplasma* (16.0 vs. 5.6%) (3). Both groups harboured similarly high levels of *Rickettsia* (96.0 vs. 100%), suggesting that transovarial transmission was highly efficient for *Rickettsia*, less efficient for *Spiroplasma* and might not occur for *Rickettsiella*. Both lower absolute numbers of bacteria including the symbionts *Spiroplasma* and *Midichloria*, and more limited diversity of bacterial species, were reported in midguts of *I. ricinus* ticks from a laboratory colony compared to wild-caught ticks, and extremely low numbers of bacteria (<100 organisms per midgut) were found in *Rhipicephalus microplus* ticks from a closed colony in Brazil (42). A bacterial symbiont, later identified as a *Cardinium* sp. (43), was isolated from first-generation adult *I. scapularis* reared in the laboratory from field-caught adults (6). *Rickettsia raoultii* was isolated from eggs laid by the first generation of adult *Dermacentor reticulatus* reared in the laboratory from field-caught ticks (21). However, we could not find any report of *in vitro* isolation of a bacterial symbiont from laboratory colony ticks maintained for additional generations.

Here we report isolation and preliminary genetic characterisation of a *Spiroplasma* from third-generation adult male and female *I. persulcatus*, originally collected in Siberia (Irkutsk Oblast, Russian Federation) and maintained in a laboratory colony for over 4 years.

## Materials and Methods

### Ticks

Unfed adult *I. persulcatus* ticks were collected from vegetation by flagging near Irkutsk, (Irkutsk Oblast, Russian Federation) at Talsy (52.024381 N, 104.657681 E) and Ust-Ordynsky (52.700295 N, 104.905164 E) in May 2015. The ticks were subsequently maintained as a laboratory colony through three generations in the tick rearing facility of the Institute of Parasitology, Biology Centre, Czech Academy of Sciences (BCCAS). All animal experiments were in accordance with the Animal Protection Law of the Czech Republic (§17, Act No. 246/1992 Sb) and with the approval of the Czech Academy of Sciences (approval no. 161/2010). All instars were fed to engorgement on guinea pigs or gerbils, incubated for moulting or oviposition at 24°C, 96% relative humidity (RH) and stored following moult or larval hatching under the same conditions. To obtain separate groups of unfed adult male and female ticks, nymphs were visually inspected following engorgement, and males were sorted from females according to their size as male nymphs are approximately one third smaller. Unfed adult male and female ticks were transferred by courier to the Tick Cell Biobank, University of Liverpool, where they were stored at 15°C, 100% RH for 19 days until used for *Spiroplasma* isolation or seven months until used for DNA extraction.

### *In vitro* Isolation of *Spiroplasma*

Five male and five female unfed adult *I. persulcatus* ticks were surface-sterilised by immersion in 0.1% benzalkonium chloride for 5 min, 70% ethanol for 1 min and 2 x 1 min rinses in sterile deionised water. After drying on sterile filter paper, the ticks were embedded in wax and their internal organs (as much as possible of midgut, salivary glands, synganglion, Malpighian tubules, rectal sac, fat body, testes/ovary) were dissected out as described previously (21). Each tick was dissected in a separate drop of Hank's balanced salt solution and the dissecting instruments were sterilised in 70% ethanol between ticks. The internal organs from each tick were inoculated into a separate culture of tick cells in a sealed, flat-sided tube (Nunc, Thermo-Fisher) and incubated at 28°C. Four embryo-derived tick cell lines were used for *Spiroplasma* isolation: *Rhipicephalus microplus* BME/CTVM23 (13) and BME26 (44), *I. ricinus* IRE11 (45) and *Ixodes scapularis* IDE2 (46). BME/CTVM23 and BME26 cells were grown in complete L-15 and L-15B media respectively, (47) and IRE11 and IDE2 cells were grown in complete L-15B300 medium (48); all media contained 100 units/ml penicillin and 100 μg/mL streptomycin. Medium was changed weekly by removal and replacement of $\frac{3}{4}$ of the medium volume and cultures were monitored by inverted microscope examination. Giemsa-stained cytocentrifuge smears were prepared as described previously (13) from all cultures on day 53 post inoculation (p.i.) and examined for presence of bacteria. All cultures were cryopreserved in vapour phase liquid nitrogen as described previously (21) on day 90 p.i.

### Molecular Characterisation of Cultured *Spiroplasma*

On day 65 p.i., the cells in each culture were resuspended and 200 μL aliquots were centrifuged at 15,000 × *g* for 5 min. DNA was extracted from the cell pellets using a DNeasy blood and tissue Mini Kit (Qiagen) following the manufacturer's instructions. DNA extracts were screened for presence of *Spiroplasma* using PCR assays amplifying fragments of the 16S rRNA (16S rRNA; ~500 bp) and RNA polymerase beta subunit (*rpoB*; ~1443 bp) genes (49, 50). Amplicons were visualised by agarose gel electrophoresis, and positive PCR products were purified using a PureLink Quick Gel Extraction and PCR Purification Combo kit (ThermoFisher) following the manufacturer's instructions and submitted for Sanger sequencing in both directions (Eurofins Genomics, Germany). Phylogenetic analyses were conducted with MEGA X using the maximum likelihood method based on the Kimura 2-parameter model and including all sites (51, 52). The nucleotide substitution model was selected according to the Bayesian information criterion (BIC) implemented in Mega X (53). Confidence values for individual branches of the resulting trees were determined by bootstrap analysis with 500 replicates. Two separate phylogenetic trees based on available 16S rRNA and *rpoB* sequences of *Spiroplasma* spp. isolated or detected in ixodid ticks were inferred. It was not possible to include all these *Spiroplasma* variants in both phylogenies because published sequences of both gene fragments amplified in this study were not available for some of them. Moreover, the *rpoB* analysis was performed with a shorter fragment (<600 bp) corresponding to the fragment available from many of these published sequences. The published sequences used in the analyses are shown in the phylogenetic trees.

### Detection of *Spiroplasma* in *I. persulcatus* Colony Ticks

DNA was extracted from 10 ticks (four male and six female) remaining from the batch shipped to Liverpool, 7 months after receipt, using a DNeasy blood and tissue Mini Kit (Qiagen) according to the manufacturer's instructions with overnight lysis. DNA was extracted from a further 17 male and 24 female ticks from the same generation maintained in the BCCAS colony, using a DNeasy blood and tissue Mini Kit (Qiagen) with the following modifications. Briefly, the ticks were homogenised individually in 200 μL of ATL buffer (Qiagen) for 2 min at 30 shakes/s in a Tissue Lyser II (Qiagen). After brief centrifugation and addition of 20 μL of proteinase K, the samples were incubated at 56°C for 30 min. The remaining steps of DNA extraction were done according to the manufacturer's instructions. To confirm species identity of the ticks screened in Liverpool, a fragment of the tick 16S rRNA gene was amplified using primer pairs 16S+1/16S-1 as described previously (54). To detect *Spiroplasma*, DNA from all ticks was PCR-screened using the specific assays for fragments of the *Spiroplasma* 16S rRNA and *rpoB* genes as described above. Randomly-selected positive amplicons were purified and sequenced as above (Liverpool ticks) or enzymatically purified using Exonuclease I FastAP and Thermosensitive Alkaline Phosphatase (ThermoFisher Scientific) and submitted for Sanger sequencing (SeqMe, Czech Republic) (BCCAS ticks), and analysed as described above.

## Results

**Figure 1:**
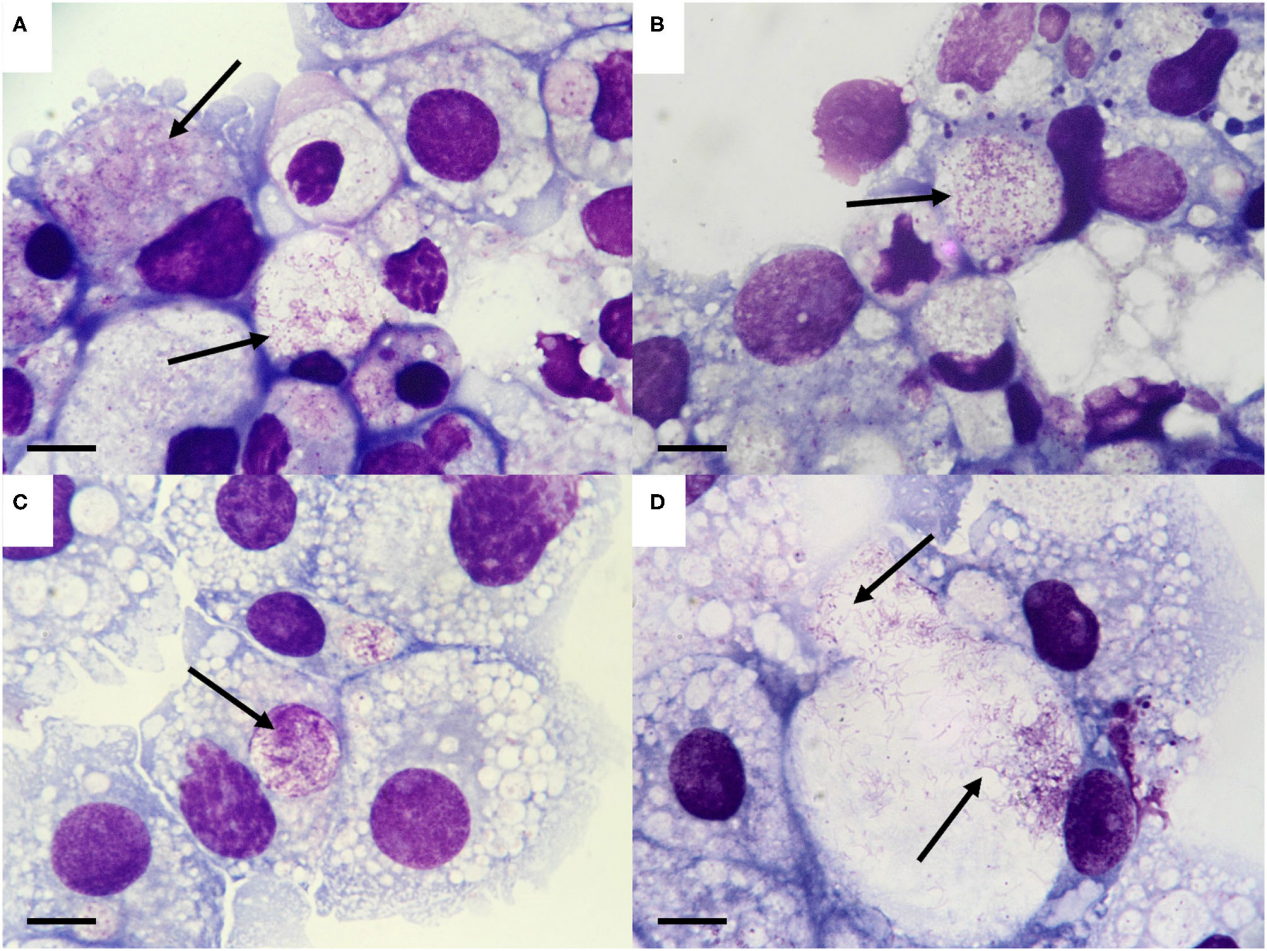
Morphology of the *Spiroplasma* sp. isolated from *Ixodes persulcatus* ticks. **(A–D)**. *Spiroplasma* (arrows) in tick cell lines inoculated with internal organs from male and female *I. persulcatus* ticks, day 53 post inoculation. **(A)** BME/CTVM23 cells inoculated with male tick #304 (*Spiroplasma* strain Irkutsk1). **(B)** BME26 cells inoculated with female tick #308 (*Spiroplasma* strain Irkutsk4). **(C,D)**. IRE11 cells inoculated with female tick #310 (*Spiroplasma* strain Irkutsk5). Giemsa-stained cytocentrifuge smears; scale bars = 10 μm.

When the tick cell cultures were examined by Giemsa-stained cytocentrifuge smear on day 53 p.i., bacteria resembling *Spiroplasma* were seen in cells that had received organs from 1/5 male and 5/5 female *I. persulcatus* ticks (Table 1). In all cases, the *Spiroplasma* were intracellular and concentrated in cytoplasmic vacuoles, but the appearance differed between the various tick cell lines (Figure 1). In the *R. microplus* cell lines (Figures 1A,B) and IDE2 (not shown), most vacuoles containing *Spiroplasma* also contained homogenous, light blue- or pink-staining background material, whereas in IRE11 cells (Figures 1C,D) such material was absent in most vacuoles containing *Spiroplasma*. It was not possible to determine whether this was due to differences between the cell lines or the *Spiroplasma* isolates, although in previous studies background material was visible in *Spiroplasma*-containing vacuoles in cells of the tick cell lines BME/CTVM23 and DALBE3 (21) but not of the tick cell lines IRE11, IRE/CTVM19 or IDE2 (20).

**Table 1:** Detection of *Spiroplasma* by microscopy and PCR analysis of tick cell lines inoculated with internal organs from male and female *Ixodes persulcatus* ticks.

**Sample no**.	**Tick gender**	**Cell line**	**Microscopy result**	***Spiroplasma*** **PCR result**		**Strain designation**
				**16S rRNA**	***rpoB***	
303	Male	BME/CTVM23	None seen	–	–	
304	Male	BME/CTVM23	*Spiroplasma*	+	+	Irkutsk1
305	Female	BME/CTVM23	*Spiroplasma*	+	+	Irkutsk2
306	Female	BME/CTVM23	*Spiroplasma*	+	+	Irkutsk3
307	Male	BME26	None seen	–	–	
308	Female	BME26	*Spiroplasma*	+	+	Irkutsk4
309	Male	IRE11	None seen	–	–	
310	Female	IRE11	*Spiroplasma*	+	+	Irkutsk5
311	Male	IDE2	None seen	–	–	
312	Female	IDE2	*Spiroplasma*	+	+	Irkutsk6

PCR amplification of fragments of the *Spiroplasma*-specific 16S rRNA and *rpoB* genes from DNA extracted on day 65 p.i. confirmed the presence of *Spiroplasma* in the six microscopically-positive cultures, and in both cases failed to amplify any products from DNA extracted from the four microscopically-negative cultures (Table 1). To determine the *Spiroplasma* infection rate in adult ticks of the parent colony, the 10 ticks remaining in Liverpool (four males, six females) and a further 41 ticks (12 males, 12 females fed as nymphs on guinea pigs and five males, 12 females fed as nymphs on gerbils) from the same generation of the BCCAS colony were screened using the *Spiroplasma* 16S rRNA and *rpoB* PCR assays. All of the ticks were positive for *Spiroplasma* by one or both assays, and amplification and sequencing of a 430 bp fragment of the tick 16S rRNA gene confirmed the species identity of the ticks tested in Liverpool as *I. persulcatus* (99.8% similarity to *I. persulcatus* from Omsk, Siberia, Russia, Genbank accession no. MH790201.1).

Sequence analysis revealed that, for the *Spiroplasma* 16S rRNA gene, all six culture isolates (designated Irkutsk1-6) and five representative tick samples screened in Liverpool were identical to each other, and identical to eight representative tick samples screened at BCCAS apart from one ambiguous nucleotide at position 105 (Table 2A). All sequences showed 99.8% similarity (99.5% query cover) to the only sequence from an *I. persulcatus*-derived *Spiroplasma* available in Genbank at the time of writing (LC388762.1) (10); interestingly, the only mismatch between our sequences and that of the Japanese isolate (10) was also at position 105 (Table 2A). The 16S rRNA sequence of the Irkutsk strains isolated from *I. persulcatus* was identical to several other *Spiroplasma* strains isolated from hard ticks: *Spiroplasma* sp. Bratislava 1 (KP967685, from Slovakian *I. ricinus*), *Spiroplasma* sp. 1033 (LC388770, from Japanese *Haemaphysalis kitaokai*) and *Spiroplasma* sp. 135 (LC388760 and LC388759, from Japanese *Ixodes monospinosus*) (10, 20) (Table 2A). Moreover, the Irkutsk 16S rRNA sequence showed 99.1–99.3% similarity to those of spiroplasmas isolated from North American *Ixodes pacificus* (*Spiroplasma ixodetis*, NR_104852), Spanish *Dermacentor marginatus* (*Spiroplasma* sp. strain DMAR11, MG859280) and Dutch *D. reticulatus* (*Spiroplasma* sp. strain DRET8, MG859282) (21, 55) (Table 2A). For the *rpoB* gene, all sequences obtained from the six culture isolates and 11 representative whole ticks were identical. At the time of writing, we could not find any published *rpoB* sequences from *I. persulcatus*-derived *Spiroplasma* for comparison, and most of those derived from other hard tick species were shorter than 600 bp. Considering the query cover higher than 99%, the *rpoB* sequence of the Irkutsk strains showed 99.9% similarity to the spiroplasmas isolated from *I. ricinus* (*Spiroplasma* sp. Bratislava1, KP967687) and *Dermacentor* spp. (*Spiroplasma* strain DMAR11, MG859278 and *Spiroplasma* strain DRET8, MG859277) (Table 2B), and 99.3% similarity to *S. ixodetis* (DQ313832). With a query cover of 43%, the sequences amplified in this study were identical to shorter sequences from spiroplasmas detected by PCR in other hard tick species (GenBank accession numbers MK267073-MK267077, MK267081-MK267085 and MK267097) (4), and also to *Spiroplasma* strain DMAR11 (MG859278) and *Spiroplasma* sp. Bratislava1 (KP967687) that showed polymorphisms in the longer gene fragment (Table 2B).

**Table 2A:** Polymorphisms in sequences detected in the *Ixodes persulcatus*-derived *Spiroplasma* sp. in this study compared to other tick-borne *Spiroplasma* spp.

***Spiroplasma* strain**	**Tick species**	**GenBank accession no**	**Positions^a^**														
			**55-56^b^**	**103**	**105**	**157**	**187**	**209**	**248**	**275**	**298**	**299**	**386**	**400**	**420**	**437**	**449-450*^b^***
*Spiroplasma* sp. Liverpool tick	*I. persulcatus*	MW498417	-	T	T	G	G	C	G	C	G	G	C	G	G	G	-
*Spiroplasma* sp. BCCAS tick	*I. persulcatus*	MW492370	-	T	K	G	G	C	G	C	G	G	C	G	G	G	-
*Spiroplasma* sp. strain Irkutsk1	*I. persulcatus*	MW498416	-	T	T	G	G	C	G	C	G	G	C	G	G	G	-
*Spiroplasma* sp. 147_ISE6	*I. persulcatus*	LC388762	-	T	**G**	G	G	C	G	C	G	G	C	G	G	G	-
*Spiroplasma* sp. 1033_C6/36	*H. kitaokai*	LC388770	-	T	**G**	G	G	C	G	C	G	G	C	G	G	G	-
*Spiroplasma* sp. 135_C6/36 & ISE6	*I. monospinosus*	LC388760, LC388759	-	T	**G**	G	G	C	G	C	G	G	C	G	G	G	-
*Spiroplasma* sp. Bratislava 1	*I. ricinus*	KP967685	-	T	**G**	G	G	C	G	C	G	G	C	G	G	G	-
*Spiroplasma ixodetis* Y32	*I. pacificus*	NR_104852	-	T	**G**	G	G	C	G	C	**A**	G	C	**A**	G	G	**G**
*Spiroplasma* sp. strain DMAR11	*D. marginatus*	MG859280	-	T	**G**	G	G	C	**R**	C	G	**R**	C	G	G	G	-
*Spiroplasma* sp. strain DRET8	*D. reticulatus*	MG859282	-	T	**G**	G	**T**	C	**A**	C	G	G	C	G	G	G	-
*Spiroplasma* sp. Hokkaido IO-1	*I. ovatus*	DQ059993	**G**	**C**	**A**	**A**	G	**T**	**A**	**T**	G	G	**A**	G	**A**	**A**	-

*Polymorphisms in the 16S rRNA gene fragment of the Spiroplasma sp. detected in whole third-generation I. persulcatus colony ticks sampled at University of Liverpool (Liverpool tick) and at the Institute of Parasitology, Biology Centre, Czech Academy of Sciences (BCCAS tick) and isolated from I. persulcatus (strain Irkutsk1) compared to other Spiroplasma strains isolated from hard tick species I. persulcatus, Ixodes monospinosus, Ixodes ricinus, Ixodes pacificus, Ixodes ovatus, Haemaphysalis kitaokai, Dermacentor marginatus and Dermacentor reticulatus*.

a *The number corresponds to the positions of nucleotide substitutions with respect to the sequences MW498416 and MW498417 amplified in this study. Corresponding base substitutions are shown. The substitutions compared to the sequences amplified in this study are shown in bold*.

b *There is an insertion between these two nucleotide bases in one sequence, a gap (-) is marked when this insertion does not occur. K = G or T; R = A or G*.

**Table 2B:** Polymorphisms in sequences detected in the *Ixodes persulcatus*-derived *Spiroplasma* sp. in this study compared to other tick-borne *Spiroplasma* spp.

***Spiroplasma* strain**	**Tick species**	**GenBank accession no**	**Positions^a^**														
			**188**	**226**	**266**	**399**	**402**	**406**	**460**	**533**	**548**	**620**	**650**	**667**	**680**	**719**	**794**
*Spiroplasma* sp. Liverpool tick	*I. persulcatus*	MW528411	C	A	C	A	A	C	G	C	A	T	G	A	A	C	A
*Spiroplasma* sp. BCCAS tick	*I. persulcatus*	MW528410	C	A	C	A	A	C	G	C	A	T	G	A	A	C	A
*Spiroplasma* sp. strain Irkutsk1	*I. persulcatus*	MW528409	C	A	C	A	A	C	G	C	A	T	G	A	A	C	A
*Spiroplasma* sp. Bratislava 1	*I. ricinus*	KP967687	**T**	A	C	A	A	**T**	G	C	A	T	G	A	A	C	A
*Spiroplasma ixodetis* Y29	*I. pacificus*	DQ313832	C	**T**	**A**	**G**	**G**	C	G	**T**	A	T	G	A	A	C	A
*Spiroplasma* sp. strain DMAR11	*D. marginatus*	MG859278	C	A	C	A	A	C	**T**	C	A	T	G	A	A	C	A
*Spiroplasma* sp. strain DRET8	*D. reticulatus*	MG859277	C	A	C	A	A	C	G	C	A	T	G	**G**	A	C	A
*Spiroplasma ixodetis* Y32^b^	*I. pacificus*	MK267069	na	na	na	na	na	na	na	**T**	A	T	G	A	A	C	A
*S. ixodetis* isolate Ixofrob^b^	*I. frontalis*	MK267074	na	na	na	na	na	na	na	C	A	T	G	A	A	C	A
*S. ixodetis* isolate IxoricR1532^b^	*I. ricinus*	MK267076	na	na	na	na	na	na	na	C	A	T	G	A	A	C	A
*S. ixodetis* isolate Ixosp1Tickpanthr11^b^	*Ixodes* sp.	MK267080	na	na	na	na	na	na	na	**T**	A	**C**	**C**	A	**T**	C	**G**
*S. ixodetis* isolate IxospT2641^b^	*Ixodes* sp.	MK267081	na	na	na	na	na	na	na	C	A	T	G	A	A	C	A
*S. ixodetis* isolate IxouriaeT2631^b^	*I. uriae*	MK267077	na	na	na	na	na	na	na	C	A	T	G	A	A	C	A
*S. ixodetis* isolate Ixoarbo2^b^	*I. arboricola*	MK267072	na	na	na	na	na	na	na	**T**	**G**	T	G	A	A	**T**	A
*S. ixodetis* isolate Ixopac2^b^	*I. pacificus*	MK267070	na	na	na	na	na	na	na	**T**	A	T	G	A	A	C	A
*S. ixodetis* isolate*Rhigeigy3^b^*	*R. geigyi*	MK267085	na	na	na	na	na	na	na	**T**	A	T	G	A	A	C	A
*S. ixodetis* isolate Rhideco^b^	*R. decoloratus*	MK267084	na	na	na	na	na	na	na	**T**	A	T	G	A	A	C	A
*S. ixodetis* isolate RhiannBSP21^b^	*R. annulatus*	MK267082	na	na	na	na	na	na	na	**T**	A	T	G	A	A	C	A
***Spiroplasma*** **strain**	**Tick species**	**GenBank accession no**	**Positions**^**a**^														
			**812**	**821**	**845**	**848**	**866**	**869**	**969**	**1022**	**1039**	**1066**	**1118**	**1313**			
*Spiroplasma* sp. (Liverpool tick)	*I. persulcatus*	MW528411	T	A	T	T	A	T	G	C	G	C	C	T			
*Spiroplasma* sp. (BCCAS tick)	*I. persulcatus*	MW528410	T	A	T	T	A	T	G	C	G	C	C	T			
*Spiroplasma* sp. strain Irkutsk1	*I. persulcatus*	MW528409	T	A	T	T	A	T	G	C	G	C	C	T			
*Spiroplasma* sp. Bratislava 1	*I. ricinus*	KP967687	T	A	T	T	A	T	G	C	G	C	C	T			
*Spiroplasma ixodetis* Y29	*I. pacificus*	DQ313832	T	A	T	T	A	T	**A**	C	G	C	C	**C**			
*Spiroplasma* sp. strain DMAR11	*D. marginatus*	MG859278	T	A	T	T	A	T	G	C	G	C	C	T			
*Spiroplasma* sp. strain DRET8	*D. reticulatus*	MG859277	T	A	T	T	A	T	G	C	G	C	C	T			
*Spiroplasma ixodetis* Y32^b^	*I. pacificus*	MK267069	T	A	T	T	A	T	**A**	C	G	C	C	na			
*S. ixodetis* isolate Ixofrob^b^	*I. frontalis*	MK267074	T	A	T	T	A	T	G	C	G	C	C	na			
*S. ixodetis* isolate IxoricR1532^b^	*I. ricinus*	MK267076	T	A	T	T	A	T	G	C	G	C	C	na			
*S. ixodetis* isolate Ixosp1Tickpanthr11^b^	*Ixodes* sp.	MK267080	**C**	**G**	**A**	**A**	**T**	T	**A**	**A**	**A**	**T**	**T**	na			
*S. ixodetis* isolate IxospT2641^b^	*Ixodes* sp.	MK267081	T	A	T	T	A	T	G	C	G	C	C	na			
*S. ixodetis* isolate IxouriaeT2631^b^	*I. uriae*	MK267077	T	A	T	T	A	T	G	C	G	C	C	na			
*S. ixodetis* isolate Ixoarbo2^b^	*I. arboricola*	MK267072	T	A	T	**C**	**T**	**C**	G	**A**	G	C	C	na			
*S. ixodetis* isolate Ixopac2^b^	*I. pacificus*	MK267070	T	A	T	T	A	T	G	**A**	G	C	C	na			
*S. ixodetis* isolate*Rhigeigy3^b^*	*R. geigyi*	MK267085	T	A	T	T	A	T	G	C	G	C	C	na			
*S. ixodetis* isolate Rhideco^*b*^	*R. decoloratus*	MK267084	T	A	T	T	A	T	G	C	G	C	C	na			
*S. ixodetis* isolate RhiannBSP21^*b*^	*R. annulatus*	MK267082	T	A	T	T	A	T	G	C	G	C	C	na			

*Polymorphisms in the rpoB gene fragment of the Spiroplasma sp. detected in the Liverpool tick and the BCCAS tick and strain Irkutsk1 compared to other Spiroplasma strains isolated from hard tick species I. ricinus, I. pacificus, D. marginatus and D. reticulatus, and sequences detected by PCR in I. pacificus, I. ricinus, Ixodes frontalis, Ixodes uriae, Ixodes arboricola, Ixodes sp., Rhipicephalus geigyi, Rhipicephalus decoloratus and Rhipicephalus annulatus*.

a *The number corresponds to the positions of nucleotide substitutions with respect to the sequences MW528409 and MW528411 amplified in this study. Corresponding base substitutions are shown*.

b *Short sequences. na: Not available. The substitutions compared to the sequences amplified in this study are shown in bold*.

**Figure 2:**
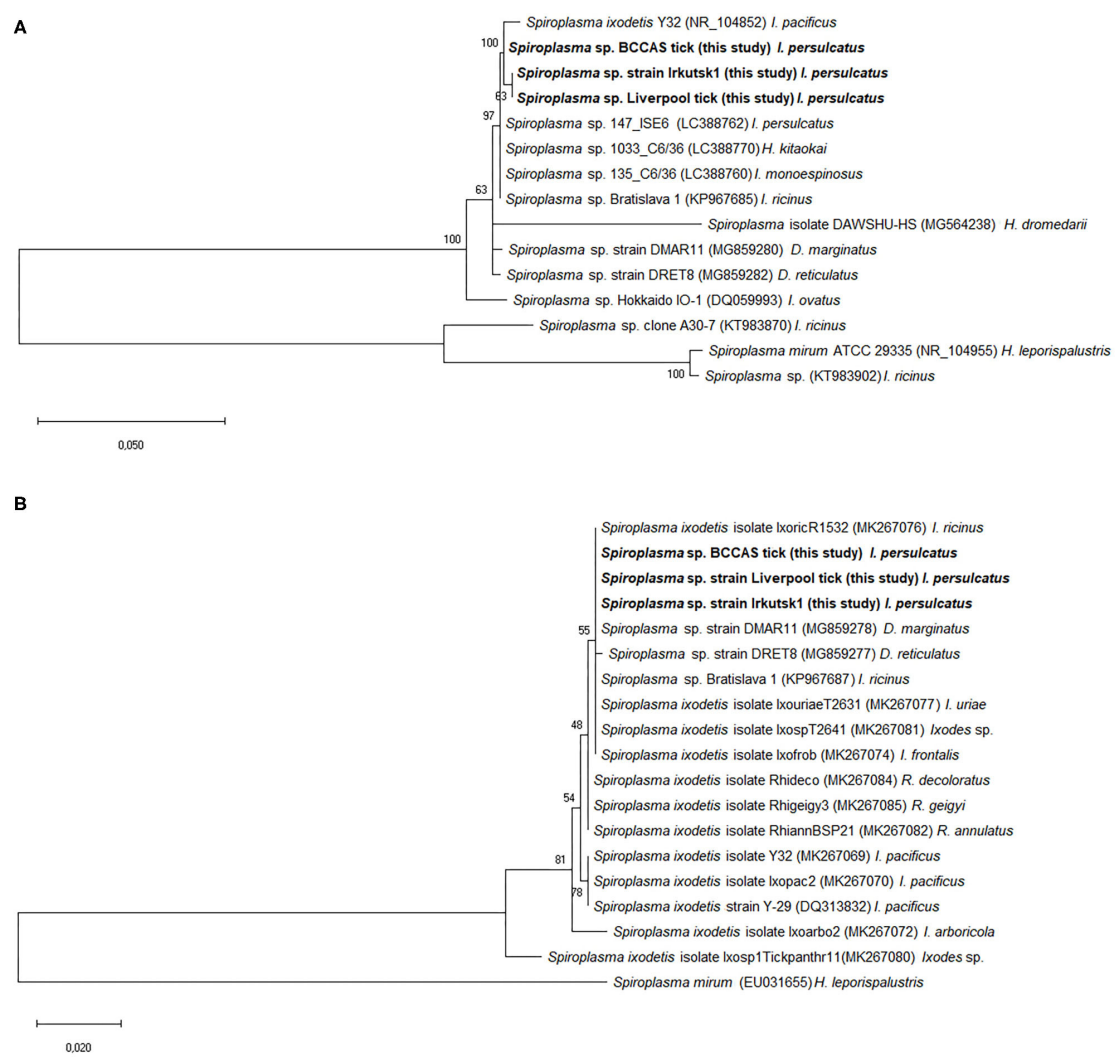
Phylogenetic analysis of *Spiroplasma* gene sequences obtained from third generation *Ixodes persulcatus* colony ticks. Sequences amplified from DNA extracted from representative adult *Ixodes persulcatus* ticks at the University of Liverpool (Liverpool tick) and the Institute of Parasitology, Biology Centre, Czech Academy of Sciences (BCCAS tick) and from a representative *Spiroplasma*-infected tick cell culture previously inoculated with internal organs from an adult *I. persulcatus* (*Spiroplasma* sp. strain Irkutsk1) were compared with published sequences from other *Spiroplasma* spp. or strains derived from ixodid tick species. The evolutionary analysis was inferred using the maximum likelihood method and Kimura 2-parameter model within the Mega X software. The trees are drawn to scale, with branch lengths measured in the number of substitutions per site. GenBank accession numbers of the sequences used in this analysis are shown in brackets following each *Spiroplasma* species or strain and before the tick species host. Sequences obtained in this study are marked in bold. **(A)** Tree constructed from 15 16S rRNA nucleotide sequences and a total of 461 positions in the final dataset. **(B)** Tree constructed from 19 *rpoB* nucleotide sequences and a total of 588 positions in the final dataset.

Phylogenetic analysis based on 16S rRNA sequences derived from *Spiroplasma* sp. strain Irkutsk1 and two representative whole ticks revealed that the *I. persulcatus* spiroplasmas clustered together with, but were not identical to, *S. ixodetis* (55) and most of the spiroplasmas from other hard ticks (Figure 2A). Similarly, the phylogeny obtained with the *rpoB* sequences showed tight clustering of the *I. persulcatus Spiroplasma* with most other tick-borne *Spiroplasma* sequences (Figure 2B).

The *Spiroplasma* 16S rRNA and *rpoB* gene sequences obtained in the present study were deposited in GenBank under accession numbers MW492370, MW498416, MW498417, MW528409-MW528411.

## Discussion

Colonisation in the laboratory has been previously reported to result in decrease or loss of the microbial symbiont *Ca*. M. mitochondrii in *I. ricinus* (2, 41), whereas *Coxiella*-like endosymbionts were detected at high prevalence in *Ornithodoros rostratus, Amblyomma americanum, Dermacentor silvarum* and *R. microplus* ticks maintained in laboratory colonies for unspecified numbers of generations (55). In the case of the *I. persulcatus Spiroplasma* in the present study, after three generations in the laboratory, 100% of whole adult ticks (21 males, 30 females) were PCR-positive for this endosymbiont. Moreover, 5/5 female ticks and 1/5 male ticks harboured sufficient levels of viable bacteria to allow *in vitro* isolation in tick cell lines. Admittedly, the sensitivity of this technique for detection of infection with *Spiroplasma* is unknown, so it is possible that the remaining four male ticks could also have harboured *Spiroplasma* but either at a level insufficient to allow isolation, or in an organ or tissue that was inadvertently not included in the inoculum, or in a state of viability not conducive to *in vitro* isolation. Tissue tropism of the symbiont *Ca*. M. mitochondrii in *I. ricinus* ticks was found to be highly specific to certain organs (56); further study is needed to determine the tissue tropism of *Spiroplasma* spp. in *Ixodes* spp. ticks.

The prevalence of *Spiroplasma* in the original Siberian field ticks from which the laboratory colony was initiated in 2015 is unknown, and therefore it is impossible to determine whether colonisation resulted in maintenance of, or increase in, the infection rate. However, it can be concluded that laboratory colonisation does not have a negative effect on occurrence of *Spiroplasma* in *I. persulcatus*, at least over three generations. There have only been two reports of detection of *Spiroplasma* in *I. persulcatus* ticks. Using 16S amplicon pyrosequencing, *Spiroplasma* were detected in salivary glands of at least 5/6 male and 5/6 female unfed *I. persulcatus* collected in the field in Japan (29), and *Spiroplasma* was successfully isolated into arthropod cell culture from 1/30 questing adult *I. persulcatus* collected in Japan (10). In contrast, no *Spiroplasma* or other mollicutes were recorded in questing *I. persulcatus* collected in the Novosibirsk area of Russia and examined by 16S metagenomic profiling (four pools of 87–120 ticks) (32), and *Spiroplasma* were not detected by species-specific 16S rRNA PCR in three questing *I. persulcatus* ticks collected in Finland (3).

*Spiroplasma* infection rates determined by molecular analysis vary widely in other *Ixodes* spp. ticks collected from the field in different geographical areas. Prevalence of *Spiroplasma* in *I. ricinus* nymphs and adults ranged from 0–0.3% in UK [(21); author's unpublished data] through 5–6% in Hungary and Czech Republic (57, 58) to 23–30% in The Netherlands, France, Switzerland and Spain (3, 59). The bacterium was detected in 10% of *Ixodes uriae* from Russia (3), 14–16% of *I. arboricola* from Belgium (3, 60) and 100% of *Ixodes ovatus* from Japan (29). The type species *S. ixodetis* was isolated from 7/30 pools representing 600 *I. pacificus* from USA, suggesting a prevalence between 1.2 and 23% (61). Considering this level of variation between species and geographical location, the infection rates of 100% in whole ticks and 60% following *in vitro* isolation in the present study suggest that *Spiroplasma* survives well-under laboratory colony conditions, in both male and female *I. persulcatus* ticks.

Presence of *Spiroplasma* in laboratory colony ticks could affect their ability to be infected experimentally with, and/or transmit, tick-borne pathogens, and therefore their use in this context. A recent study examined correlations between presence of *Spiroplasma* in field-collected *I. ricinus* in Switzerland, and presence in these ticks of bacterial pathogens and symbionts (40). Negative correlations were found between *Spiroplasma* and the pathogens *Rickettsia* spp. and *Borrelia valaisiana* in individual *I. ricinus*, but positive correlations were found between *Spiroplasma* and the symbionts *Lariskella* and *Rickettsiella* at the population level. Further studies are needed to examine whether presence of *Spiroplasma* in *Ixodes* spp. ticks has any effect on acquisition, replication or transmission of tick-borne arboviruses such as TBEV or protozoa such as *Babesia* spp., or indeed any effect on the viability of the ticks themselves.

The molecular analysis revealed almost no differences between the *Spiroplasma* isolated from colonised *I. persulcatus* of Russian origin and cultured for 2.5 months in cell lines derived from heterologous tick species (*I. ricinus, I. scapularis* and *R. microplus*), *Spiroplasm*a DNA detected in whole ticks from the same colony and the *Spiroplasma* isolated into *I. scapularis* cells from Japanese *I. persulcatus* (10). Ambiguity was seen in a single nucleotide in the ~476 bp fragment of the 16S rRNA gene amplified in the present study, and the same nucleotide showed a difference when compared with the sequence from the Japanese isolate. The *rpoB* gene fragment analysed in our study was longer than the 16S rRNA sequences, providing more phylogenetic information, although the shorter length of most of the published sequences from other tick species (4) reduced the coverage available for comparison. The overall topology of the tree and the relationship between strains in the tick-borne *Spiroplasma* branch were very close to the results based on the 16S rRNA gene, although neither of these gene fragments are sufficient to confidently separate *Spiroplasma* strains or species. Nevertheless, as reported previously (21) it is clear that the spiroplasmas harboured by different *Ixodes* spp. ticks are not identical, and also differ from those harboured by *Dermacentor* spp. ticks from broadly contiguous geographic regions.

In conclusion, our study has shown that efficient vertical transmission of *Spiroplasma* can be maintained in *I. persulcatus* ticks under laboratory colony conditions for at least three generations, and has confirmed that co-cultivation of internal organs with tick cell lines is a simple and effective technique for *in vitro* isolation of intracellular tick symbionts such as *Spiroplasma* spp. Further molecular analysis of the cultured *Spiroplasma* strains derived from *I. persulcatus*, either by Sanger sequencing of additional genes or by whole genome sequencing, is required to clarify the phylogenetic relationships between them and *Spiroplasma* harboured by *I. persulcatus* of different geographical origins and by other tick species, and to facilitate an accurate taxonomic classification of these genotypes.

## Data Availability Statement

The original sequences generated for this study are publicly available in the NCBI Genbank repository under accession numbers MW492370, MW498416, MW498417, MW528409-MW528411.

## Ethics Statement

The animal study was reviewed and approved by Czech Academy of Sciences (approval no. 161/2010).

## Author Contributions

AB, VH, JE, TV, MP, and LB-S carried out the experimental work. AB, VH, AP, and LB-S analysed the data. JC, IK, and DR carried out the field work. LB-S conceived the study and drafted the manuscript. AB, VH, JC, DR, and AP revised the manuscript. All authors reviewed and agreed to the final version.

## Conflict of Interest

The authors declare that the research was conducted in the absence of any commercial or financial relationships that could be construed as a potential conflict of interest.
